# Orbit Angular Momentum MIMO with Mode Selection for UAV-Assisted A2G Networks

**DOI:** 10.3390/s20082289

**Published:** 2020-04-17

**Authors:** Tao Hu, Yang Wang, Bo Ma, Jie Zhang

**Affiliations:** 1 School of Communication and Information Engineering, Chongqing University of Posts and Telecommunications, Chongqing 400065, China; hutao@stu.cqupt.edu.cn (T.H.); jie.zhang@sheffield.ac.uk (J.Z.); 2 Department of Electronic and Electrical Engineering, The University of Sheffield, Sheffield S10 2TN, UK; bma4@sheffield.ac.uk

**Keywords:** line-of-sight (LOS), air-to-ground (A2G), unmanned aerial vehicle (UAV), orbit angular momentum (OAM), branch and bound search-based mode selection (BBS-MS), spectrum efficiency (SE)

## Abstract

As an emerging solution for line-of-sight (LOS) wireless communications, in air-to-ground (A2G) channels, the unmanned aerial vehicle (UAV), and allowing the dynamic and flexible network deployments enables the supplement or/and replacement of the terrestrial base stations (BSs). However, in conventional multiple-input-multiple-output (MIMO) systems, high-speed communications are significantly limited by channel crosstalks and spectrum scarcities. An orbit angular momentum (OAM) wireless network, allowing co-existence of multiple physical channels within the same frequency band, offers new degrees of freedom to address this dilemma. In this paper, we investigate the UAV-based A2G radio vortex wireless networks and study its channel model. Then we propose a branch and bound search-based mode selection (BBS-MS) scheme, which uses the spatial distribution characteristics of vortex beams to optimize the spectrum efficiency (SE). Theoretical derivations and numerical results demonstrate that our developed BBS-MS scheme can obtain the optimal performance, which outperforms conventional OAM-based MIMO systems. Also, it possesses a lower complexity compared with exhaustive searches.

## 1. Introduction

Orbit angular momentum (OAM) is a physical property of electromagnetic waves, which can be used to generate multiple orthogonal vortex beams [1]. Recently, OAM multiplexing has attracted much attention as a novel approach for improving the spectral efficiency (SE) of multiple-input-multiple-output (MIMO) wireless communication systems [2,3,4]. The first experimental test has demonstrated that OAM can be applied to wireless communications [2]. Then, a radio vortex-multiple-input multiple-output (RV-MIMO) system with high SE is introduced in [3], where multiple spiral phase plate (SPP) antennas are used for OAM generations. Also, the feasibility that the uniform circular array (UCA) antenna can be used to creat twist beams was theoretically and practically verified [4]. Furthermore, OAM techniques with the characteristic of orthogonal transmissions are widely used in optical communications [5,6], line-of-sight (LOS) communications [7,8,9], and radar imaging [10,11].

In the case of natural or human-made disasters, the traditional terrestrial communication networks will be damaged and communication links can be destroyed. The flexible and low complexity air base stations (BSs) can be rapidly established to provide in-time communication supports in this situation [12,13,14]. The unmanned aerial vehicle with its flexibility and low complexity was considered to be a candidate of the air BS. The unmanned aerial vehicle (UAV) with its flexibility and low complexity is viewed as a candidate of the air BS. Besides, the UAV techniques are widely used in atmospheric sciences [15,16,17,18,19,20,21,22,23]. The authors in [15] presented the information about the use of rotary-wing droned to sample near-surface fields. In [16], a UAV-based system was proposed to measure the profiles and turbulence in the boundary layer. Moreover, some measurements relative to the wind and turbulence in the boundary layer were carried out in [17,18,19]. The researchers in [20] proposed a method for detecting atmospheric Lagrangian coherent structures using a fixed-wing drone. Then, a coordinated drone and ground-based weather measurement system was introduced to predict Lagrangian structures [21]. Besides, the UAV can be used to validate the weather modeling [22] and monitoring trace tropospheric gases [23].

**Figure 1:**
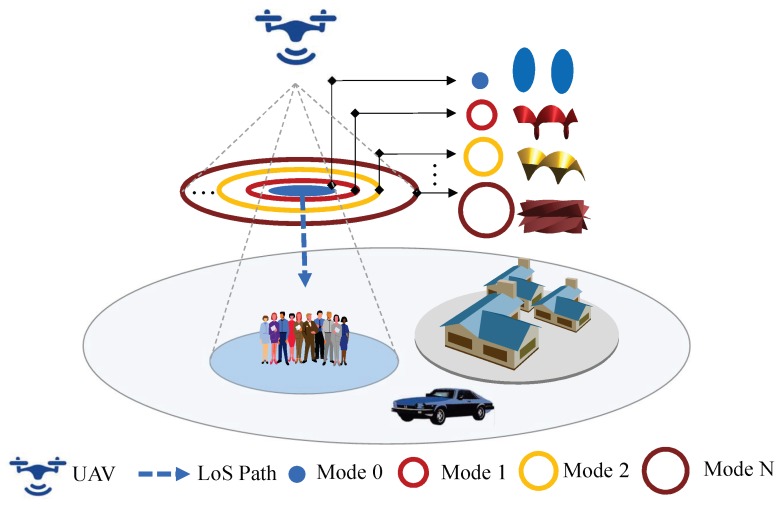
The scenario of the UAV-based radio vortex wireless network.

Meahwhile, in air-to-ground (A2G) wireless networks, UAV-based BS (UAV-BS) plays a fundamental role in serving temporal hotspots to improve the quality of service [24,25]. In addition, UAV-BS also plays a crucial role in enabling reliable and high-speed wireless backhaul connectivity for the traditional terrestrial networks [26]. Temporal hotspots such as big tournaments, open-air concerts, and huge gatherings, where users tend to be fixed or less mobile, often yield severe network congestion. Fortunately, in such scenarios, reliable and ideal LOS communication links can be established to improve the quality and capacity of communications by optimally placing drones to avoid obstacles. Also, OAM-based MIMO can significantly improve the SE of A2G network due to the dominant LOS channels in A2G communication links. Generally, both of the scenarios need timely nodes for assistance but still suffer from accompanying interference and lack of frequency bands. According to the physical property of Laguerre Gauss (LG) beams, the energy distribution of OAM waves is in the shape of a doughnut [1,9] as shown in Figure 1. As the mode absolute value increases, the angle between maximum radiation direction and propagation direction becomes extensive. It is notable that the circles shown in Figure 1 denote the range of the power distribution which coresponding to the OAM main lobes. Thus, any terrestrial users equipped with a general OAM receiving antenna cannot receive the whole OAM signal ultimately.

To improve the SE of the A2G wireless communication links, we first model the UAV-based LOS A2G channel that makes mode division multiplexing (MDM) transfer possible. Then we continue with a design of the mode selection scheme, named by branch and bound search-based mode selection (BBS-MS), touching upon the improvements in performance and the reductions of computational complexities. Also, we derive capacity expressions for the mode selection system, and the numerical simulations demonstrate the superiority of BBS-MS scheme over conventional MDM systems is obtained.

## 2. System Model and Problem Formulation

In this section, we consider an air-to-ground-radio vortex (A2G-RV) wireless communication system, as shown in Figure 1. At the transmitter, the drone is equipped with an $N_{t}$-element UCA antenna, where $T_{X_{n}}$ ($1\leq n\leq N_{t}$) is $n^{\mathrm{th}}$ transmit antenna element, the parameter α is the transmitting rotation angle, and $2\pi /N_{t}$ is the angle between adjacent antenna elements. However, at the receiver, the ground user is equipped with a 2-element OAM receiver ($R_{X_{1}}$ and $R_{X_{2}}$), where φ denotes the receiving azimuth angle, and the angle between two receiving antenna elements is denoted by δ. Please note that the OAM signal can be precisely captured once the constant $B<\frac{\pi }{|l_{\max}|}$, where $l_{\max}$ is the maximum value of the OAM topological charge [27]. The parameter $d_{n1}$ $\left(d_{n2}\right)$ denotes the distances between $n^{\mathrm{th}}$ (n∈[1,N]) transmitting element and the receiving antenna $R_{X_{1}}$ $\left(R_{X_{2}}\right)$. The altitudes of the UAV-based OAM transmitter with the radius of *R* and the ground user are $h_{t}$ and $h_{r}$, respectively.

Also, A2G-RV wireless channels experience the dominant LOS channels even in dense urban environments, which is different from the conventional terrestrial communications [14,24,25]. Therefore, the LOS A2G channel matrix with multi-path, denoted by $H\in C^{N_{r}\times N_{t}}$, which can be expressed as

(1) $$H=H_{\mathrm{LOS}}$$

where $H_{\mathrm{LOS}}$ is the LOS channel matrix from the transmitter to the receiver.

### 2.1. The LOS A2G Channel

**Figure 2:**
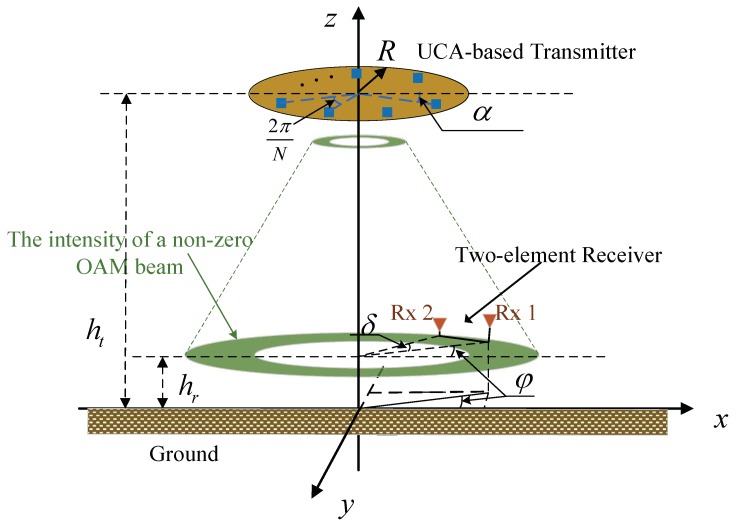
The LOS geometric channel model.

To facilitate channel modeling, as depicted in Figure 2, we employ the cylindrical coordinate system (r,θ,ξ) to express the position of the antenna element, in which *r*, θ, and ξ denote the polar axis, angular coordinate and longitudinal axis, respectively. Furthermore, the position of the centre of the transmit UCA $T_{X_{n}}$ is given by (0,0,0), and the positions of the receive antenna $R_{X_{1}}$ and $R_{X_{2}}$ are expressed by $\left(r,\varphi ,h_{t}-h_{r}\right)$ and $\left(r,\varphi +\delta ,h_{t}-h_{r}\right)$, respectively, where *r* is the radial distance of the receive antenna pair corresponding to the propagation distance as $h_{t}-h_{r}$ in the longitudinal axis. Thus, the direct distances from $n^{\mathrm{th}}$ transmitting element to the receiving elements $R_{X_{1}}$ and $R_{X_{2}}$ can be calculated as follows

(2) $$d_{\mathrm{LOS}}\left(n,1\right)=\sqrt{a_{n1}^{2}+b_{n1}^{2}+{\tilde{d}}_{n1}^{2}}$$

(3) $$d_{\mathrm{LOS}}\left(n,2\right)=\sqrt{a_{n2}^{2}+b_{n2}^{2}+{\tilde{d}}_{n2}^{2}}$$

with ${\tilde{d}}_{n1}={\tilde{d}}_{n2}=h_{t}-h_{r}$ and

(4) $$a_{nm}=\{\begin{array}{cc} r\cos\varphi -R\cos(\phi_{n}+\alpha ), & m=1\\ r\cos\left(\varphi +\delta \right)-R\cos\left(\phi_{n}+\alpha \right), & m=2 \end{array}$$

(5) $$b_{nm}=\{\begin{array}{cc} r\sin\varphi -R\sin(\phi_{n}+\alpha ), & m=1\\ r\sin\left(\varphi +\delta \right)-R\sin\left(\phi_{n}+\alpha \right), & m=2 \end{array}$$

where $\phi_{n}=2\pi n/N_{t}$ denotes the basic azimuth angle for the transmitting UCA. Thus, the $d_{\mathrm{LOS}}\left(n,1\right)$ and $d_{\mathrm{LOS}}\left(n,2\right)$ can be rewritten as follows

(6) $$\begin{array}{c} d_{\mathrm{LOS}}\left(n,1\right)\overset{\left(a\right)}{\approx }\sqrt{h_{t}^{2}+h_{r}^{2}+r^{2}+R^{2}}-\frac{h_{t}h_{r}}{\sqrt{h_{t}^{2}+h_{r}^{2}+r^{2}+R^{2}}}-\frac{rR\cos(\phi_{n}+\alpha -\varphi )}{\sqrt{h_{t}^{2}+h_{r}^{2}+r^{2}+R^{2}}} \end{array}$$

(7) $$\begin{array}{c} d_{\mathrm{LOS}}\left(n,2\right)\overset{\left(a\right)}{\approx }\sqrt{h_{t}^{2}+h_{r}^{2}+r^{2}+R^{2}}-\frac{h_{t}h_{r}}{\sqrt{h_{t}^{2}+h_{r}^{2}+r^{2}+R^{2}}}-\frac{rR\cos(\phi_{n}+\alpha -\varphi -\delta )}{\sqrt{h_{t}^{2}+h_{r}^{2}+r^{2}+R^{2}}} \end{array}$$

where (a) denotes the total sum of squares approximation with the example of $\sqrt{a^{2}-2b}\approx a-\frac{b}{a}$.

In free-space LOS scenarios, there is an only direct path at both ends of the transceiver, and the propagation of electromagnetic waves leads to amplitude corruption and periodic phase change of the transmitted signal. Therefore, the channel responses between $n^{\mathrm{th}}$ antenna element of the transmitting UCA and $m^{\mathrm{th}}$ antenna element of the receiving UCA is denoted by [3]

(8) $$h_{\mathrm{LOS}}\left(n,m\right)=\frac{\beta \lambda }{4\pi d_{\mathrm{LOS}(n,m)}}e^{-jkd_{\mathrm{LOS}(n,m)}}$$

where β is the attenuation corresponding to the pattern of the antenna element, $d_{\mathrm{LOS}}\left(n,m\right)$ is the direct distance from $n^{\mathrm{th}}$ transmitting element to $m^{\mathrm{th}}$ receiving element, λ denotes the wavelength of the carrier wave, and k=2π/λ denotes the wavenumber. Consequently, in the A2G radio vortex communication system, the channel responses between $n^{\mathrm{th}}$ antenna element in the transmitting UCA and the receiving antenna $R_{X_{1}}$ and $R_{X_{2}}$ can be rewritten as

(9) $$\begin{array}{c} h_{\mathrm{LOS}}\left(n,1\right)\overset{\left(b\right)}{\approx }\frac{\beta \lambda }{4\pi {\tilde{d}}_{\mathrm{LOS}}}e^{-jk{\tilde{d}}_{\mathrm{LOS}}}e^{\frac{jk[h_{t}h_{r}+rR\cos\left(\phi_{n}+\alpha -\varphi \right)]}{{\tilde{d}}_{\mathrm{LOS}}}} \end{array}$$

(10) $$\begin{array}{c} h_{\mathrm{LOS}}\left(n,2\right)\overset{\left(b\right)}{\approx }\frac{\beta \lambda }{4\pi {\tilde{d}}_{\mathrm{LOS}}}e^{-jk{\tilde{d}}_{\mathrm{LOS}}}e^{\frac{jk[h_{t}h_{r}+rR\cos\left(\phi_{n}+\alpha -\varphi -\delta \right)]}{{\tilde{d}}_{\mathrm{LOS}}}} \end{array}$$

with ${\tilde{d}}_{\mathrm{LOS}}=\sqrt{h_{t}^{2}+h_{r}^{2}+r^{2}+R^{2}}$, where (b) denotes the approximate condition that $d_{\mathrm{LOS}}\left(n,1\right)=d_{\mathrm{LOS}}\left(n,2\right)\approx \sqrt{h_{t}^{2}+h_{r}^{2}+r^{2}+R^{2}}$ for the amplitude term.

### 2.2. A2G-RV Channel

In the UCA-based multiple mode transmitter, the vortex beams can be generated by feeding the same input symbol $s_{l}$ to each antenna element in transitting UCA with a successive phase different. Consequently, the transmitted signal at $n^{\mathrm{th}}$ antenna element, denoted by $x_{n}$, is given as

(11) $$\begin{array}{c} x_{n}=\frac{1}{\sqrt{N_{t}}}\sum_{l\in L}\rho_{l}s_{l}e^{j[\phi_{n}+\alpha ]l} \end{array}$$

where $L=\left\{l_{0},l_{1},\ldots ,l_{i},\ldots ,l_{N_{t}-1}\right\}$ is the set of the OAM modes with the value of $l_{i}\in \left[-N_{t}/2,N_{t}/2\right)$, $\rho_{l}$ is the transmitted power allocated to $l^{\mathrm{th}}$ OAM mode, and $\phi_{n}+\alpha$ is the transmitted azimuthal angle. Therefore, the matrix expression of the complex baseband signal mode of the A2G-RV scheme would be described as

(12) $$\begin{array}{c} y=Hx+w \end{array}$$

where $y\in C^{M\times 1}$ is the received vector, $H\in C^{M\times N_{t}}$ is the A2G channel matrix, $w\in C^{M\times 1}$ is the vector of the received additive complex white Gaussian noise (AWGN) with zero-mean and variance $\sigma^{2}$, and the $x\in C^{N_{t}\times 1}=F\mathrm{Ps}$, where $F\in C^{N_{t}\times N_{t}}$ is the discrete Fourier transform (DFT) matrix, $P=\mathrm{diag}\left\{[\rho_{0}\;\rho_{1}\;\cdots \;\rho_{N_{t}-1}]\right\}$ is the transmitting power allocation matrix, and $s={\left[s_{0}\;s_{1}\;\cdots \;s_{N_{t}-1}\right]}^{T}$ is the data symbols vector.

In the proposed A2G-RV system, the channel responses between $n^{\mathrm{th}}$ transmit antenna, and the receive antenna $R_{X_{1}}$ and $R_{X_{2}}$ have the equations as

(13) $$\begin{array}{c} h_{\mathrm{LOS}}^{l}\left(n,1\right)=\frac{{\tilde{\beta }}_{l,1}\lambda }{4\pi {\tilde{d}}_{\mathrm{LOS}}}e^{-jk{\tilde{d}}_{\mathrm{LOS}}}e^{-j(\varphi -\phi_{n}-\alpha )l}e^{\frac{jk[h_{t}h_{r}+rR\cos\left(\phi_{n}+\alpha -\varphi \right)]}{{\tilde{d}}_{\mathrm{LOS}}}} \end{array}$$

(14) $$\begin{array}{c} h_{\mathrm{LOS}}^{l}\left(n,2\right)=\frac{{\tilde{\beta }}_{l,2}\lambda }{4\pi {\tilde{d}}_{\mathrm{LOS}}}e^{-jk{\tilde{d}}_{\mathrm{LOS}}}e^{-j(\varphi +\delta -\phi_{n}-\alpha )l}e^{\frac{jk[h_{t}h_{r}+rR\cos\left(\phi_{n}+\alpha -\varphi -\delta \right)]}{{\tilde{d}}_{\mathrm{LOS}}}} \end{array}$$

where ${\tilde{\beta }}_{l,1}$ and ${\tilde{\beta }}_{l,2}$ denote the attenuation outside of the circle regions of OAM beams. The attenuation coefficients satisfy ${\tilde{\beta }}_{1}={\tilde{\beta }}_{2}=\beta$ when the two-element receive antenna locates in the circle regions of OAM beams. In this case, the fading of OAM signals obeys the Friis Law. However, in a general A2G-RV communication system, the receiver (ground user) could be outside of the OAM circle regions, where the attenuation coefficients ${\tilde{\beta }}_{l,1}$ and ${\tilde{\beta }}_{l,2}$ depend on the location of the receiver. Furthermore, in the proposed system, the attenuation coefficients satisfy ${\tilde{\beta }}_{l,1}={\tilde{\beta }}_{l,2}={\tilde{\beta }}_{l}$ due to the equal radial distance *r* of each receive element. The following will present the mathematical derivation of ${\tilde{\beta }}_{l}$.

The Laguerre-Gaussian (LG) beams, the most widely used example of OAM research, were considered to describe the radio OAM beams [27]. By solving the partial differential equation ∂ulp∂Il∂r∂r=0, where $u_{lp}$ is the expression of the LG beams [27], the radius of the maximum beam intensity can be given by

(15) $$r_{\max,l}\left(z\right)=\sqrt{\frac{\left|l\right|}{2}}w_{l}\left(z\right)=w_{l}\sqrt{\frac{\left|l\right|}{2}\left(1+{\left(\frac{z}{z_{R}}\right)}^{2}\right)}$$

Given the A2G-RV mode selection wireless communication system, the response between $n^{\mathrm{th}}$ transmit antenna and the receive antenna ${R_{X}}_{m}$, and the response between $n^{\mathrm{th}}$ transmit antenna and the receive antenna ${R_{X}}_{m^{\prime }}$ can be denoted by [27]

(16) $$\begin{array}{c} u_{l}\left(n,m\right)=h_{\mathrm{LOS}}^{l}\left(n,m\right)\hat{x} \end{array}$$

(17) $$\begin{array}{c} u_{l}\left(n,m^{\prime }\right)=h_{\mathrm{LOS}}^{l}\left(n,m^{\prime }\right)\hat{x} \end{array}$$

where $u_{l}$ is the expression of LG beams [8,27], $\hat{x}$ is the unit pulse input, ${R_{X}}_{m}$ and ${R_{X}}_{m^{\prime }}$ donate the receiver are inside and outside of the OAM regions, respectively. Based on this assumption, the positions of the receive antenna ${R_{X}}_{m}$ and ${R_{X}}_{m^{\prime }}$ are expressed by $\left(r_{\max,l}\left(z\right),\varphi ,h_{t}-h_{r}\right)$ and $\left(r_{nm^{\prime }},\varphi ,h_{t}-h_{r}\right)$. Furthermore, and the responses can be derived by using Equations (13) and (14) as follows

(18) $$\begin{array}{c} u_{l}\left(n,m\right)={\tilde{\beta }}_{l}\frac{{\left(\sqrt{2}r_{\max,l}\left(z\right)\right)}^{\left|l\right|}}{w_{l}{\left(z\right)}^{|l|+1}}e^{\frac{-r_{\max,l}^{2}\left(z\right)}{w_{l}^{2}\left(z\right)}}e^{\frac{-jkr_{\max,l}^{2}\left(z\right)}{2R_{l}\left(z\right)}}e^{-j(|l|+1)}e^{jl\varphi_{m}} \end{array}$$

(19) $$\begin{array}{c} u_{l}\left(n,m^{\prime }\right)={\tilde{\beta }}_{l}\frac{{\left(\sqrt{2}r_{nm^{\prime }}\right)}^{\left|l\right|}}{w_{l}{\left(z\right)}^{|l|+1}}e^{\frac{-r_{nm^{\prime }}^{2}}{w_{l}^{2}\left(z\right)}}e^{\frac{-jkr_{nm^{\prime }}^{2}}{2R_{l}\left(z\right)}}e^{-j(|l|+1)}e^{jl\varphi_{m}^{\prime }} \end{array}$$

Combining Equations (13)–(19), the attenuation ${\tilde{\beta }}_{l,1}$ can be calculated as

(20) $$\begin{array}{c} {\tilde{\beta }}_{l,m^{\prime }}=\beta \frac{d_{\mathrm{LOS}}\left(n,m^{\prime }\right)}{d_{\mathrm{LOS}}\left(n,m\right)}{\left(\frac{r_{nm^{\prime }}}{r_{\max,l}\left(z\right)}\right)}^{\left|l\right|}e^{-\frac{r_{nm^{\prime }}^{2}-r_{\max,l}^{2}\left(z\right)}{w_{l}^{2}\left(z\right)}}e^{-\frac{jk(r_{nm^{\prime }}^{2}-r_{\max,l}^{2}\left(z\right))}{2R_{l}\left(z\right)}}e^{jk[d_{\mathrm{LOS}}\left(n,m^{\prime }\right)-d_{\mathrm{LOS}}\left(n,m\right)]} \end{array}$$

where $r_{nm^{\prime }}$ is the radial distance between $n^{\mathrm{th}}$ transmit antenna and the receive antenna ${R_{x}}_{m^{\prime }}$. When the receiver locates inside of the circle region corresponding to OAM mode *l*, the attenuation coefficientsatisfies ${\tilde{\beta }}_{l,m^{\prime }}=\beta$. Substitute Equation (20) into Equation (13), the channel responses can be rewritten as

(21) $$\begin{array}{c} h_{\mathrm{LOS}}^{l}\left(n,m^{\prime }\right)=\frac{\beta \lambda }{4\pi {\tilde{d}}_{\mathrm{LOS}}}{\left(\frac{r_{nm^{\prime }}}{r_{\max,l}\left(z\right)}\right)}^{\left|l\right|}e^{-\frac{r_{nm^{\prime }}^{2}-r_{\max,l}^{2}\left(z\right)}{w_{l}^{2}\left(z\right)}}e^{-\frac{jk(r_{nm^{\prime }}^{2}-r_{\max,l}^{2}\left(z\right))}{2R_{l}\left(z\right)}}e^{\frac{jk[h_{t}h_{r}+rR\cos\left(\phi_{n}+\alpha -\varphi_{m}\right)]}{{\tilde{d}}_{\mathrm{LOS}}}}e^{-jk{\tilde{d}}_{\mathrm{LOS}}}e^{-j(\varphi_{m}-\phi_{n}-\alpha )l} \end{array}$$

Combining Equations (11), (12) and (21), the A2G-RV channel matrix, denoted by $H_{\mathrm{RV}}$ which can be expressed as follows

(22) $$\begin{array}{c} H_{\mathrm{RV}}=\left[\begin{array}{ccc} h_{11} & \cdots & h_{1N_{t}}\\ h_{21} & \cdots & h_{2N_{t}}\\ \vdots & \ddots & \vdots \\ h_{M1} & \cdots & h_{MN_{t}} \end{array}\right]\left[\begin{array}{ccc} 1 & \cdots & 1\\ 1 & \cdots & e^{j\Phi_{2N_{t}}l_{N_{t}-1}}\\ \vdots & \ddots & \vdots \\ 1 & \cdots & e^{j\Phi_{MN_{t}}l_{N_{t}-1}} \end{array}\right] \end{array}$$

where the entry $h_{mn}$ can be replaced by $h_{\mathrm{LOS}}\left(n,m\right)$, and $\Phi_{mn}=\phi_{n}+\alpha -\varphi_{m}$ is the azimuth angle.

## 3. Capacity and Mode Selection

### 3.1. Exact Upper Bounds

In this paper, we suppose that the channel gains related to the transmit modes is known at the transmitter, and the independent Gaussian information symbols are transmitted. Furthermore, we consider a time division duplexing (TDD) MIMO system, where only uplink channels need to be estimated due to channel reciprocity. The capacity can be written as [28]

(23) $$\begin{array}{c} C=\log_{2}\left[\det\left(I_{N_{t}}+\frac{P_{T}}{N_{t}}H_{\mathrm{RV}}H_{\mathrm{RV}}^{H}\right)\right] \end{array}$$

where $P_{T}$ is the total transmitted power, and $H_{\mathrm{RV}}=\left[h_{1},\ldots ,h_{n},\ldots ,h_{N_{t}}\right]$ is the A2G-RV channel matrix. Please note that the uniform power allocation policy is considered in this paper for simplicity, where the transmitting power allocated to each effective OAM mode is $\rho =\frac{P_{T}}{L}$ with $1\leq L\leq N_{t}$. With the channel gains at the transmit side, we can sort the channel gains $\left|\right|h_{n}{\left|\right|}^{2}$ in descending order, i.e., $\left|\right|h_{1}{\left|\right|}^{2}\geq \left|\right|h_{2}{\left|\right|}^{2}\geq \cdots \geq \left|\right|h_{N_{t}}{\left|\right|}^{2}$. Then, the transmitter can obtain the best index subset $S_{L}=\left\{1,\ldots ,L\right\}$, which determines the best *L* out of $N_{t}$ transmit OAM modes from the ordered index set $S=\left\{1,\ldots ,N_{t}\right\}$ by using a rate-limited return channel [28]. Finally, the optimal transmit channel matrix relative to the optimal subset $S_{L}$, denoted by ${\tilde{H}}_{\mathrm{RV}}=\left[{\tilde{h}}_{1},\ldots ,{\tilde{h}}_{L}\right]$, can be obtained.

Based on the description above, the mode selections for maximizing the channel capacity can be built into an optimization problem as follows

(24) $$\begin{array}{c} {\tilde{H}}_{\mathrm{opt}}=\underset{{\tilde{H}}_{\mathrm{RV}}\in H}{\arg\;\;\max}\;\{\log_{2}\left[\det\left(I_{L}+\rho {\tilde{H}}_{\mathrm{RV}}^{H}{\tilde{H}}_{\mathrm{RV}}\right)\right]\} \end{array}$$

(25) $$\mathrm{Subject}\;\mathrm{to}\left\{\begin{array}{c} \rho L\leq P_{T}\\ \rho \geq 0\\ 1\leq L\leq N_{t} \end{array}\right.$$

where H is the sets of total $C(N_{t},L)$ possible submatrices for the mode selection system. Thus, the capacity of the mode selection system is derived as

(26) $$\begin{array}{c} C_{\mathrm{opt}}=\log_{2}\left[\det\left(I_{L}+\rho {\tilde{H}}_{\mathrm{opt}}^{H}{\tilde{H}}_{\mathrm{opt}}\right)\right] \end{array}$$

Benefit from the orthogonality between different OAM subchannels, the upper bound on the capacity for the A2G-RV MIMO system with mode selection can be defined as [28]

(27) $$\begin{array}{c} C_{A2G-\mathrm{RV}}^{\mathrm{Upper}}=\sum_{l=1}^{L}\log_{2}\left(1+\rho \gamma_{l}\right) \end{array}$$

with $\gamma_{l}=\frac{|h_{l}{|}^{2}}{\sigma_{l}^{2}}$, where $h_{l}$ is the A2G-RV channel amplitude gain, which was derived in Equation (21) for LOS path, and $\sigma_{l}^{2}$ is the variance of the received complex AWGN which corresponding to the OAM mode *l*.

Given an ideal A2G-RV communication system, we can accurately derive the upper bound on the mode selection channel capacity for the LOS path. In this case, the expression of the capacity equation is a closed-form. However, in practical applications, the channel capacity may be variable as well as the random channel matrix $H_{\mathrm{RV}}$. To the best of our knowledge, there is no statistics model of RV-MIMO channels so far. In this paper, we focus on the superiority of the mode selection scheme over the conventional MDM scheme in the ideal OAM channel rather than in the statistical model.

### 3.2. The Branch and Bound Search for Mode Selection

In the proposed mode selection system, the number of the selected transmit OAM modes should satisfy the constraint that $L\leq \min(N_{t},M)$. The OAM mode selection problem given in Equation (24), where several best OAM subchannels are chosen for transmission, which is similar to the antenna selection problem [29]. Both the mode selection and antenna selection problem can be classified as a feature subset selection problem. Based on this similarity, we can directly apply many optimized search algorithms used by the antenna selection to the mode selection. In terms of algorithm selection, exhaustive search and greed search are the two most popular algorithms for antenna selection and beam selection [29,30]. On the one hand, exhaustive search provides the optimal performance as well as tremendous computational cost, especially with a massive MIMO system [30]. On the other hand, a greedy search that relies on its ultra-low complexity has been widely applied in beam/antenna selection systems. Therefore, the choice of algorithm mainly depends on the system configurations. To address this problem, we proposed a fast and optimal searching algorithm, named by branch and bound search-based mode selection (BBS-MS), which can also be used in a large-scale system. Based on the optimization problem in Equation (24), furthermore, we employ the minimum loss channel capacity to construct the criterion function for the BBS-MS algorithm, and the definition of the minimum loss channel capacity will be given in the following content.

It is well known that the global optimal BBS scheme is very efficient without exhaustive search once the corresponding monotonic feature function can be constructed [31]. Based on the searching direction, the BBS scheme can be classified into upward and downward searching algorithms [32]. In a UCA-based short-distance OAM wireless communication system, we certainly expect to choose as many as possible OAM subchannels for transmission. For mode selection in this paper, the downward BBS algorithm is selected, where the maximum value of a monotonically decreasing criterion function needs to be found out. Specifically, the procedure of designing a criterion function can be divided into three steps as follows

#### 3.2.1. Selection of the Sub-Criterion Function

We introduce a loss capacity function, which composed of the removed $N_{t}-L$ subset to be the sub-criterion function due to the search principle of the downwards BBS algorithm. Please note that the downwards BBS algorithm starts with a full set and then discards one OAM mode resource per step from this full set until there is only an *L* feature subset left.

Let ${\tilde{H}}_{n}\in C^{M\times \bar{n}}$ be the sub-channel matrix of $H_{\mathrm{RV}}$ relative to $\bar{n}=N_{t}-n$ best OAM modes by discarding *n* worst OAM modes. Thus, the loss capacity function is given by $C_{\mathrm{loss}}\left(n\right)=C-\log_{2}\left[\det\left(I_{M}+\tilde{\rho }{\tilde{H}}_{n}{\tilde{H}}_{n}^{H}\right)\right]$ with $\tilde{\rho }=P_{T}/N_{t}$. Suppose that in the n+1 step of OAM mode discard, the sub-channel ${\tilde{h}}_{j}$ corresponding to $j^{\mathrm{th}}$ column of $H_{\mathrm{RV}}$ is discarded, which yields to a minimum contribution to the capacity function. The new sub-channel matrix ${\tilde{H}}_{n+1}\in C^{M\times \bar{n+1}}$ can be obtained by dropping ${\tilde{h}}_{j}$ from ${\tilde{H}}_{n}$. Then, it has

(28) $$\begin{array}{c} C_{\mathrm{loss}}\left(n+1\right)=\log_{2}\left[\frac{C}{\det(I_{M}+\tilde{\rho }{\tilde{H}}_{n+1}{\tilde{H}}_{n+1}^{H})}\right] \end{array}$$

with ${{\tilde{H}}_{n+1}{\tilde{H}}_{n+1}}^{H}={\tilde{H}}_{n}{\tilde{H}}_{n}^{H}-{\tilde{h}}_{j}{\tilde{h}}_{j}^{H}$ and $C=\det(I_{N_{t}}+\tilde{\rho }H_{\mathrm{RV}}H_{\mathrm{RV}}^{H})$. By applying the Sherman-Morrison formula [28] to Equation (28), we obtain the transformation

(29) $$\begin{array}{cc} C_{\mathrm{loss}}\left(n+1\right) & =\log_{2}\left[\frac{C}{\det(I_{M}+\tilde{\rho }{\tilde{H}}_{n}{\tilde{H}}_{n}^{H})}\right]-\log_{2}\left[\frac{\det(I_{M}+\tilde{\rho }{\tilde{H}}_{n+1}{\tilde{H}}_{n+1}^{H})}{\det(I_{M}+\tilde{\rho }{\tilde{H}}_{n}{\tilde{H}}_{n}^{H})}\right]\\ & =C_{\mathrm{loss}}\left(n\right)-\log_{2}\left[\det\left(I_{M}-\frac{\tilde{\rho }{\tilde{h}}_{j}{\tilde{h}}_{j}^{H}}{I_{M}+\tilde{\rho }{\tilde{H}}_{n}{\tilde{H}}_{n}^{H}}\right)\right]\\ & =C_{\mathrm{loss}}\left(n\right)-\log_{2}\left(1-\tilde{\rho }h_{\mathrm{RV},j}^{H}B_{n}h_{\mathrm{RV},j}\right)\\ & =C_{\mathrm{loss}}\left(n\right)-\log_{2}\left(1-\tilde{\rho }\epsilon_{j,n}\right)\\ & =C_{\mathrm{loss}}\left(n\right)-\nabla_{j,n} \end{array}$$

where $B_{n}={\left(I_{M}+{\tilde{H}}_{n}{\tilde{H}}_{n}^{H}\right)}^{-1}$ and $\epsilon_{j,n}={\tilde{h}}_{j}B_{n}{\tilde{h}}_{j}^{H}$ are notations, suppose that the mode index $J_{n}$, which can minimize $C_{\mathrm{loss}}^{n+1}$ in Equation (29), was found in n+1 step. Then, the updated matrix inverse $B_{n+1}$ can be expressed as

(30) $$\begin{array}{c} B_{n+1}={\left(I_{M}+{\tilde{H}}_{n+1}{\tilde{H}}_{n+1}^{H}\right)}^{-1}=B_{n}+b_{n+1}^{H}b_{n+1} \end{array}$$

where $b_{n+1}=\frac{B_{n}{\tilde{h}}_{J_{n}}}{\sqrt{{\tilde{\rho }}^{-1}+\epsilon_{J_{n}}}}$.

#### 3.2.2. Construction of the Criterion Function

The loss capacity function in Equation (29) is monotonically increasing due to $\nabla_{j,n}<0$. In this case, we introduce an offset to reverse the monotonicity of loss capacity function $C_{\mathrm{loss}}\left(n\right)$ which is similar to the scheme in [32], and then the criterion function can be defined as

(31) $$\begin{array}{c} {\tilde{C}}_{\mathrm{loss}}\left(n\right)=\sum_{s=0}^{n-1}\Lambda_{s}-C_{\mathrm{loss}}\left(n\right) \end{array}$$

where $\Lambda_{s}=-\log_{2}\left(1-\frac{\tilde{\rho }\gamma_{s}^{2}}{I_{M}+\tilde{\rho }{\tilde{H}}_{s+1}{\tilde{H}}_{s+1}^{H}}\right)$ is the offset, and $\gamma_{s}=\min_{j\in I_{s}}\;||{\tilde{h}}_{j}{\left|\right|}_{F}$ for the mode selections, where $I_{s}$ is the mode set of all of the candidate mode sources in $s^{\mathrm{th}}$ step for discarding the worst mode. In the following, we need to prove that ${\tilde{C}}_{\mathrm{loss}}\left(n\right)$ is a monotonically decreasing function of *n*.

#### 3.2.3. Proof of Monotonicity

Combining Equations (29) and (31), the recursive form of ${\tilde{C}}_{\mathrm{loss}}\left(n\right)$ is given by

(32) $$\begin{array}{c} {\tilde{C}}_{\mathrm{loss}}\left(n+1\right)={\tilde{C}}_{\mathrm{loss}}\left(n\right)+\Lambda_{n}+\nabla_{j,n} \end{array}$$

with $n=0,1,\ldots ,N_{t}-L-1$. From Equation (32), we need to prove that $\Lambda_{n}+\nabla_{j,n}\leq 0$, which ensures ${\tilde{C}}_{\mathrm{loss}}\left(n+1\right)$ is a monotonically decreasing function of *n*. In other words, we need to verify that $\Lambda_{n}\leq \left|\nabla_{j,n}\right|$.

**Lemma** **1.** *Given $\nabla_{j,n}=\log_{2}\left(1+\tilde{\rho }\epsilon_{j,n}\right)$ in Equation (29) and $\Lambda_{n}$ in Equation (31). Then we have $\Lambda_{n}\leq \left|\nabla_{j,n}\right|,\;n=0,1,\ldots ,N_{t}-L-1$.*

**Proof.**  Thanks to a similar justification in [32]. From Equations (29) and (30), we have

(33) $$\epsilon_{j,n}=\left\{\begin{array}{c} \frac{{\tilde{h}}_{j}{\tilde{h}}_{j}^{H}}{I_{M}+\tilde{\rho }H_{\mathrm{RV}}H_{\mathrm{RV}}^{H}}\\ {\tilde{h}}_{j}\left(\frac{1}{I_{M}+\tilde{\rho }H_{\mathrm{RV}}H_{\mathrm{RV}}^{H}}+\sum_{s=1}^{n}b_{s}^{H}b_{s}\right){\tilde{h}}_{j}^{H} \end{array}\right.$$

where n=0 and $n=1,2,\ldots ,N_{t}-L-1$ are the conditions for equality in Equation (33).

When n=0, we have

(34) $$\begin{array}{c} |\nabla_{j,0}\left|=\right|\log_{2}\left(1-\tilde{\rho }\epsilon_{j,0}\right)|\\ =|\log_{2}\left(1-\frac{\tilde{\rho }{\tilde{h}}_{j}{\tilde{h}}_{j}^{H}}{I_{M}+\tilde{\rho }H_{\mathrm{RV}}H_{\mathrm{RV}}^{H}}\right)|\\ \overset{\left(a\right)}{\geq }\left|\log_{2}\left(1-\frac{\tilde{\rho }\gamma_{s}^{2}}{I_{M}+\tilde{\rho }{\tilde{H}}_{n+1}{\tilde{H}}_{n+1}^{H}}\right)\right|=\Lambda_{0} \end{array}$$

where the “=” in the condition (a) holds when the candidate mode index $j\in I_{s}$ in 0^th^ discard step.

When $n=1,2,\ldots ,N_{t}-L-1$. The matrix $\sum_{s=1}^{n}b_{s}^{H}b_{s}$ is a positive semi-definite matrix since the gram matrix $b_{s}^{H}b_{s}$ is positive semi-definite. Then

(35) $$\begin{array}{c} B_{n}-\frac{1}{I_{M}+\tilde{\rho }H_{\mathrm{RV}}H_{\mathrm{RV}}^{H}}=\sum_{s=1}^{n}b_{s}^{H}b_{s}\geq 0 \end{array}$$

Thus, we have

(36) $$\begin{array}{c} |\nabla_{j,n}\left|=\right|\log_{2}\left(1-\tilde{\rho }\epsilon_{j,0}\right)|\\ =|\log_{2}\left[1-\tilde{\rho }{\tilde{h}}_{j}\left(\frac{1}{I_{M}+\tilde{\rho }H_{\mathrm{RV}}H_{\mathrm{RV}}^{H}}+\sum_{s=1}^{n}b_{s}^{H}b_{s}\right){\tilde{h}}_{j}^{H}\right]|\\ \overset{\left(b\right)}{\geq }\left|\log_{2}\left(1-\frac{\tilde{\rho }\gamma_{s}^{2}}{I_{M}+\tilde{\rho }{\tilde{H}}_{n+1}{\tilde{H}}_{n+1}^{H}}\right)\right|=\Lambda_{n} \end{array}$$

where the “=” in the condition (b) holds when the candidate mode index $j\in I_{s}$ in $n^{\mathrm{th}}$ discard step. □

Please note that the criterion function ${\tilde{C}}_{\mathrm{loss}}\left(n\right)$ is a monotonically decreasing function of *n*, which equals to the offset $\sum_{s=0}^{n-1}\Lambda_{s}$ minus the sub-criterion function $C_{\mathrm{loss}}\left(n\right)$ according to Equation (31). Moreover, the selected mode subset yielding a minimum value of $C_{\mathrm{loss}}\left(n\right)$ will produce the globally best value of ${\tilde{C}}_{\mathrm{loss}}\left(n\right)$ since the offset $\sum_{s=0}^{n-1}\Lambda_{s}$ is a constant value in each channel realization and independent of the selected mode subset. The optimal mode subset leading to the maximum value of the criterion function ${\tilde{C}}_{\mathrm{loss}}\left(n\right)$ can be found by using the proposed branch and bound algorithm.

#### 3.2.4. Searching Procedure

Thanks to the pseudocode of BAB-search-based beam selection in [24], the proposed BBS-MS scheme is shown in Algorithm 1. It is notable that if the initial value of *B* is close to the optimal ${\tilde{C}}_{\mathrm{loss}}^{\mathrm{opt}}\left(N_{t}-L\right)$, lesser nodes need be evaluated than that with a smaller *B*, which leads to a dependable *B*. For simplicity, we choose the initial value as B=−∞ in this paper.

**Figure 3:**
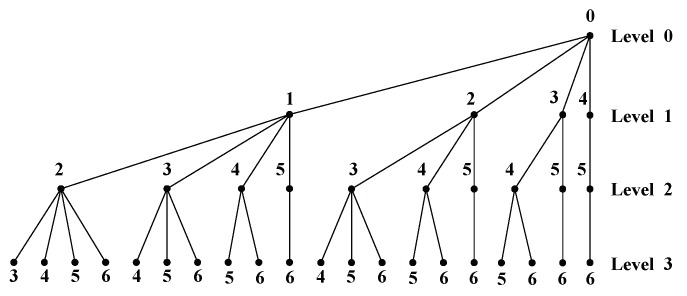
Illustration of the BBS-MS searching tree with $N_{t}=8$ and L=3.

To better understand the downward BBS-MS algorithm, Figure 3 shows an example of searching trees by discarding 3 out of 8 OAM modes. In addition, the main steps of the BBS-based searching can be summarized as follows

**Step 0: Initialize the parameters**. Set $S=\left\{1,\ldots ,N_{t}\right\}$, $S^{\prime }=\left\{1,2,\cdots ,N_{t}-L\right\}$, $S_{\mathrm{loss}}=\emptyset$, $N_{0,1}=L+1$, $J_{0}=1$, B=−∞, and n=1;

**Step 1: Initialize S**. Compute ${\tilde{C}}_{\mathrm{loss}}\left(n\right)$ for all OAM subchannels corresponding to the OAM mode set S and record the values of all ${\tilde{C}}_{\mathrm{loss}}\left(n\right)$ into the set j in descending order.

**Step 3: Check the lower-bound.** If ${\tilde{C}}_{\mathrm{loss}}\left(n\right)<B$, return the entry *J* to the set $S^{\prime }$ and shift to Step 4. In addition if $n=N_{t}-L-1$, shift to Step 5. Or else, let n=n+1 and then shift to Step 1.

**Step 4: Backtrack.** Let n=n+1. If $n=N_{t}-L$, terminate the BBS-MS algorithm. Or else, return the entry *J* to the set $S^{\prime }$ and shift to Step 2.
**Algorithm 1** The BBS-MS scheme**Require:**1:Initialization parameters: $B=\frac{1}{I_{M}+\tilde{\rho }H_{\mathrm{RV}}H_{\mathrm{RV}}^{H}}$, B=−∞, ${\tilde{C}}_{\mathrm{loss}}\left(i\right)=M$, n=0, $J_{0}=1$, $S=\left\{1,2,\ldots ,N_{t}\right\}$, $S^{\prime }=\left\{1,2,\cdots ,N_{t}-L\right\}$, and the discared mode index set $S_{\mathrm{loss}}=\emptyset$2:$\nu_{j}=\left|\right|{\tilde{h}}_{j}{\left|\right|}^{2},\forall j\in S$3:$\epsilon_{j}={\tilde{h}}_{j}^{H}B{\tilde{h}}_{j},\forall_{j}\in S$4:$\gamma_{s}=\min_{j\in I_{s}}\;\nu_{j},\forall s\in S^{\prime }$5:$\Lambda_{s}=-\log_{2}\left(1-\frac{\tilde{\rho }\gamma_{s}^{2}}{I_{M}+\tilde{\rho }{\tilde{H}}_{s+1}{\tilde{H}}_{s+1}^{H}}\right),\forall s\in S^{\prime }$6:**if**$n=N_{t}-L-1$ **then**7: ${\tilde{C}}_{\mathrm{loss}}{\left(n+1\right)}_{j}={\tilde{C}}_{\mathrm{loss}}\left(n\right)+\nabla_{j,n}+\Lambda_{N_{t}-L-1},\forall_{j}\in I_{n}$8: **if**
$\min_{s\in I_{N_{t}-L-1}}\;{\tilde{C}}_{\mathrm{loss}}{\left(n+1\right)}_{s}<B$
**then**9:  $J=\underset{s\in I_{N_{t}-L-1}}{\arg\;\max}\;{\tilde{C}}_{\mathrm{loss}}{\left(n+1\right)}_{s}$10:  update $S_{\mathrm{loss}}=S_{\mathrm{loss}}\cup \left\{J\right\}$11:  $B=\max_{s\in I_{N_{t}-L-1}}\;{\tilde{C}}_{\mathrm{loss}}{\left(n+1\right)}_{s}$12: **end if**13:**else**14: ${\tilde{C}}_{\mathrm{loss}}{\left(n+1\right)}_{j}={\tilde{C}}_{\mathrm{loss}}\left(n\right)+\nabla_{j,n}+\Lambda_{n},\forall_{j}\in I_{n}$15: sort ${\tilde{C}}_{\mathrm{loss}}{\left(n+1\right)}_{j},\forall_{j}\in I_{n}$ in a desend order to get an ordered index set j16: $\bar{B}=B,{\bar{\nu }}_{j}=\nu_{j},\forall_{j}\in S$17: **for**
$i=1:|I_{n}|$
**do**18:  $J={\left[j\right]}_{i}$19:  **if**
${\tilde{C}}_{\mathrm{loss}}{\left(n+1\right)}_{j}>B$
**then**20:   $K=\left\{{\left[j\right]}_{i+1},{\left[j\right]}_{i+2},\cdots ,{\left[j\right]}_{N_{t}-n+1}\right\}$21:   update the index set $S_{\mathrm{loss}}=S_{\mathrm{loss}}\cup \left\{J\right\}$22:   $b_{n+1}=\frac{B_{n}{\tilde{h}}_{J_{n}}}{\sqrt{{\tilde{\rho }}^{-1}+\epsilon_{J}}}$, and $B=\bar{B}+b_{n+1}^{H}b_{n+1}$23:   ${\tilde{C}}_{\mathrm{loss}}\left(n\right)={\tilde{C}}_{\mathrm{loss}}{\left(n+1\right)}_{J}$24:   $\epsilon_{s}={\tilde{h}}_{s}^{H}\left({\tilde{\epsilon }}_{s}+b_{n+1}^{H}b_{n+1}\right){\tilde{h}}_{s},\forall s\in K$25:   $\nabla_{s}=\log_{2}\left(1-\tilde{\rho }\epsilon_{s}\right),\forall s\in K$26:   n=n+1, go to line 627:  **else**28:   break the loop29:  **end if**30: **end for**31:**end if**32:**Output:** the final mode index set $S_{\mathrm{selected}}=S\backslash S_{\mathrm{loss}}$

**Step 5: Update the lower-bound.** Let $B=\max_{s\in I_{N_{t}-L-1}}\;{\tilde{C}}_{\mathrm{loss}}{\left(n+1\right)}_{s}$, where ${\tilde{C}}_{\mathrm{loss}}\left(n+1\right)$ is obtained by discarding OAM modes $1,\cdots ,N_{t}-L$ and then save it as the temporary optimal solution. Return the entry *J* to the set $S_{\mathrm{loss}}$ and shift to Step 4.

## 4. Numerical Results

In this section, we evaluate the performance of the proposed BBS-MS, the greedy search, the exhaustive search, and the conventional MDM schemes. First, we analyze the ability of OAM waves corresponding to different mode number for information transmission when considering different locations of receivers. Then, we evaluate the spectrum efficiency of the proposed BBS-MS scheme considering the different numbers of selected transmitting modes. The default simulation parameters are summarized in Table 1. Notably, the standard value of the available *L* is default as 2 in this paper due to the same performance between the two modes that are negative to each other.

**Figure 4:**
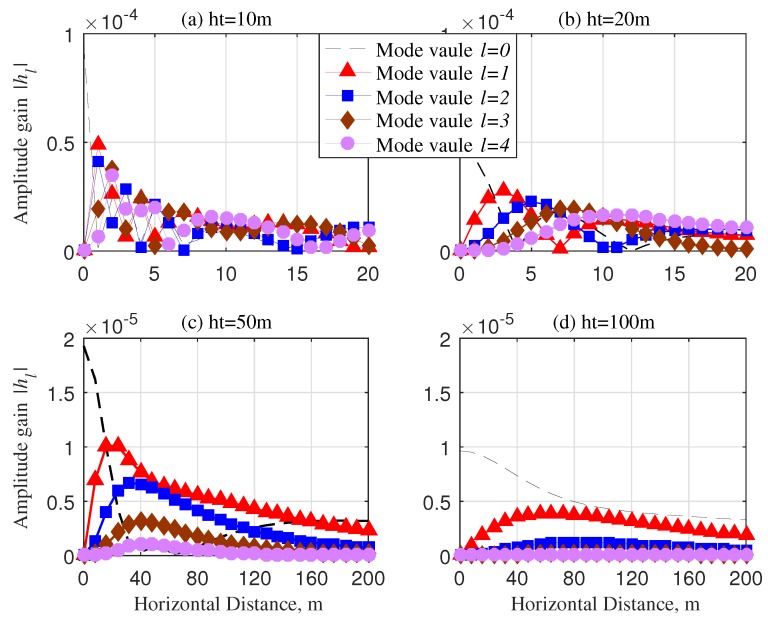
Amptitude gain $|h_{l}|$, SNR = 20 dB and R=20λ: (**a**) $h_{t}=10\;m$; (**b**) $h_{t}=20\;m$; (**c**) $h_{t}=50\;m$; (**d**) $h_{t}=100\;m$.

Figure 4 delineates the amplitude gains relative to the different locations of receivers. As illustrated in Figure 4a, within a relatively short transmission range, all OAM waves almost possess the same amplitude gain with the horizontal distance increasing. However, in Figure 4b, we can observe that the OAM waves with different mode numbers consecutively obtain the optimal amplitude gain in the process of the receiver away from the centre point. With the altitude of UAV increasing, both in Figure 4c,d, this phenomenon is more visible, and the amplitude gains of the high-order OAM waves are close to zero. The result also validates that the fundamental field distribution property of vortex electromagnetic waves, i.e., concentric zone pattern.

**Figure 5:**
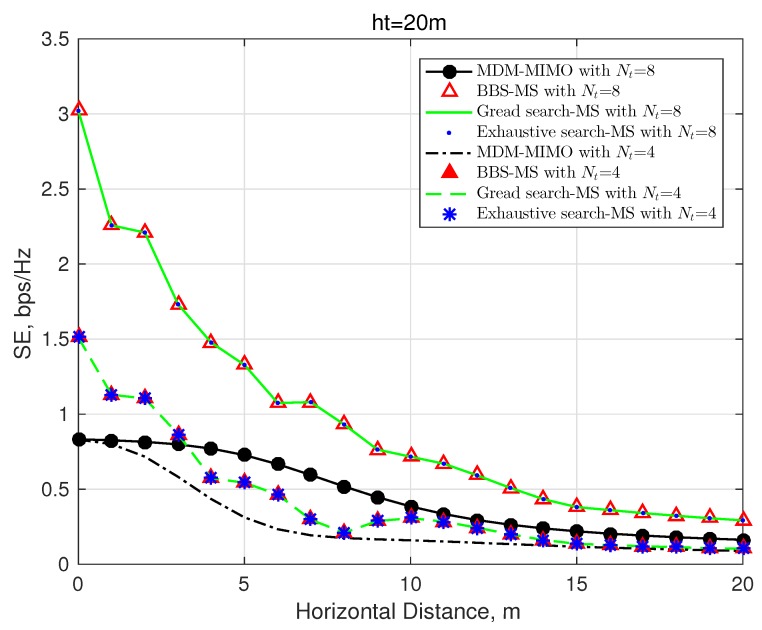
Spectrum efficiency, R=20λ, SNR = 20 dB, and L=2.

In Figure 5, we evaluate the performance of the traditional MDM system and other mode selected-based schemes, including the proposed BBS-MS, the greedy search, and the exhaustive search algorithms. Notably, both the MDM and the conventional MIMO systems possess the same performance in the LOS channel. Since the sub-optimal greedy search can achieve the most optimal performance in such a small-scale mode selection system, we can observe that the three mode selected algorithms obtain the same performance, for both $N_{t}=8$ and $N_{t}=4$ cases. Also, in the similar conventional antenna selection systems, the same conclusion was drawn in [12]. Since the properties of divergence and circular radiant field of OAM waves, the received intensities emitted by different OAM signals depend on the location of the receiver. In other words, In a medium or long distance OAM wireless network, only a part of OAM modes are suitable for transmission. Thus, the full mode MDM system obtains the worst performance.

**Figure 6:**
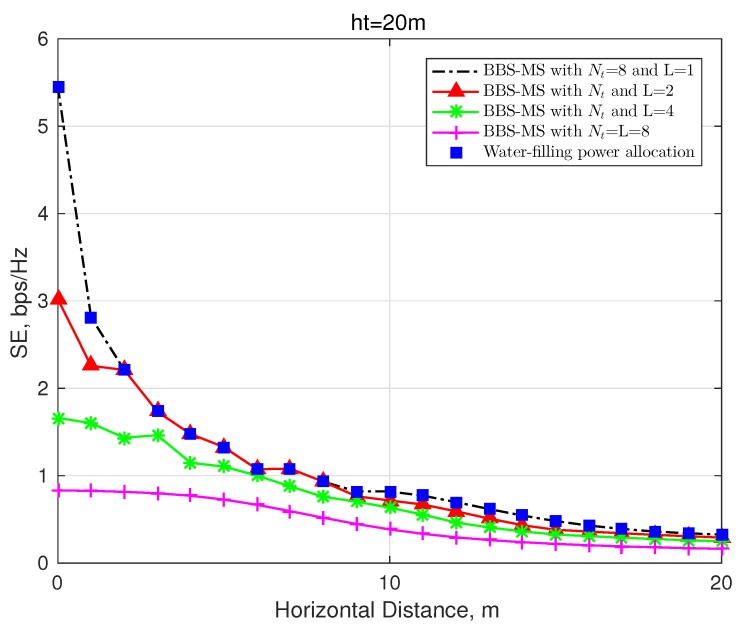
Spectrum efficiency, R=20λ and SNR = 20 dB.

The evaluation of the different numbers of the selected OAM modes is presented in Figure 6, where we set $h_{t}=20$ m, SNR = 20 dB, and R=10λ and employ the uniform power allocation scheme for comparison. It can be observed that the smaller the number of selected modes *L*, the better SE enhancement the mode selection system can obtain over the MDM systems. As is well know, the optimal system performance can be achieved by adopting a water-filling power allocation strategy. Due to different transmission gains of OAM waves in wireless channels, there is a most energetic OAM beam, which enables the mode selection scheme to obtain the optimal system performance. That is to say, a single-mode with the best transmission gain can be selected to maximize the SE.

## 5. Conclusions

In this paper, by reviewing the major field distribution of electromagnetic vortex waves, we explored the mode selected-based radio vortex communication system in the A2G scenario. To maximize the SE of the system, the BBS-MS was proposed for OAM mode selections, which can obtain the optimal performance with low computational complexity and is suitable for large-scale OAM systems. By analyzing the simulation results, we found that the mode reuse technique does not offer additional SE for single user OAM wireless communications. Thus, we made a conclusion that this orthogonal transfer technology only really delivers performance gains when multiple data transmissions simultaneously and multi-user environments are considered. Besides, in our future work, more practical conditions or even experience tests need to be considered for the theoretical verification.

## Figures and Tables

**Table 1:** Simulation Parameters.

Parameters	Values
The number of transmitting antennas	$N_{t}=8$
The number of receiving antennas	M=2
The number of the available modes	L=2
The relative rotation angle	α=0
The radius of the transmitter array	R=20λ
The transmission SNR	SNR = 20 dB
The altitude of the ground user	$h_{r}=1.5$ m
The altitude of the drone	$h_{t}=20$ m
The centre frequency of carrier wave	f=70 GHz
